# The Bridge Building Model: connecting evidence-based practice, evidence-based research, public involvement and needs led research

**DOI:** 10.1186/s40900-021-00320-y

**Published:** 2021-10-30

**Authors:** Heidi Ormstad, Gro Jamtvedt, Ida Svege, Sally Crowe

**Affiliations:** 1 University of South-Eastern Norway, Drammen, Norway; 2 Oslo Metropolitan University, Oslo, Norway; 3 Crowe Associates, Oxford, UK

**Keywords:** Evidence-based practice, Evidence-based research, Public involvement, Needs led research

## Abstract

This paper describes a model developed by an interdisciplinary team of research and public engagement specialists, with backgrounds in health and social care research, higher education, evidence-based practice, leadership, commissioning research and public involvement and engagement. The model we propose combines *evidence-based practice*, *evidence-based research*, *public involvement* and *needs led research*. Our aim is to capitalise on the joining of the rationale and methods for these approaches, which have all been highlighted as important, but for which there has been a lack of models for integration. Our ambition is to argue for and show an effective and evidence-based way of working that bridges health and social care needs identification, evidence-based practice and research.

## Plain English Summary

This paper describes a model that combines different concepts in healthcare research that the authors suggest belong together. The concepts are; Evidence Based Practice, where healthcare is based on the best research evidence, Evidence Based Research, where new research is developed in the context of what research already exists, public involvement in research, where service users, carers and health and social care professionals engage with research, and Needs Led Research, where new research addresses the needs of people that use healthcare services.

Combining these important approaches in our model is new and we argue for and show a way of working that ‘builds bridges’ between health and social care needs, healthcare practice and research.

The Bridge Building Model has been developed by a team of research and public involvement specialists, with a variety of backgrounds in health and social care research. These include education, healthcare leadership using evidence in healthcare and research funding.

## Background

Evidence-based practice was derived from evidence-based medicine (EBM), which was first introduced by Gordon Guyatt [1]. Both approaches contend with decisions about health care, and that such should be based on the best evidence, knowledge of those providing care and last but not least, the preferences of those receiving care [2].

Evidence-based research (EBR) as a concept and movement was introduced to highlight the problem of researchers who don’t systematically and transparently refer to the totality previous research when justifying and developing new research projects. In short, health research should build systematically on previous research [3, 4].

The mismatch between the research preferences and priorities of patients and clinicians on one hand and those of researchers on the other hand, has encouraged more patient and public involvement in research in general and in research priority setting [5].

In this paper we emphasize what we call “Needs Led Research” (NLR). Needs Led Research is a term agreed by the author team to explain the research outputs of a ‘Bridge Builder’ research process. This approach implicates that new research should be designed to answer *both quality assured evidence gaps, as well as the needs and priorities from users and society*. The model and approach will increase the relevance and usefulness of health research.

Evidence-based practice, evidence-based research, public involvement and needs led research have all been highlighted as important. In order to capitalise on the joining of the rationale and methods for these approaches, they can be integrated, and we have developed a “Bridge Building Model”.

## Main text

In order to make sense of the Bridge Building Model that connects evidence-based practice, evidence-based research, patient and public involvement and needs led research, we explain each component part in more detail.

### Evidence-based practice (EBP)—high quality research into clinical practice

The term evidence-based medicine (EBM) was first introduced by Gordon Guyatt [1], when he recommended that physicians should search for, critically assess and summarize clinical research, as well as assessing the relevance of the research. This would advance the inclusion of updated evidence into clinical practice. David Sackett described EBM as: *“The practice of evidence-based medicine means integrating individual clinical expertise with the best available external clinical evidence from systematic research. By individual clinical expertise we mean the proficiency and judgment that we individual clinicians acquire through clinical experience and clinical practice”* [6]. The term EBM was widely adopted to describe an approach to decision-making that spread at practically every level of health care, as well as other fields, such as social and environmental contexts (Campbell Collaboration and Centre for Environmental Evidence).

From EBM flows evidence-based practice (EBP), where decisions about health care should be based on the best available, current, valid and relevant evidence. These decisions should be made by those receiving care, informed by the tacit and explicit knowledge of those providing care, within the context of available resources [2]. The intent of EBP is that empirical research should inform clinical practice such as, prevention, treatment and rehabilitation, as well as clinical guidelines and education. To implement EBP, clinicians must have access to “best practice” or recommendations based on updated evidence and have the communication tools (e.g., listening skills) to establish patient preferences.

Lin and co-workers have reviewed feasible steps of EBP and interventions to overcome common barriers, such as lack of time, resources, or training to locate and appraise research studies [7]. The concept of EBP has continuously developed since the introduction, increasingly emphasising quality of evidence (GRADE) and shared decision making into it [8]. EBP can mean different things to different people. The authors see it as clinical practice and health and social care that is based on reliable evidence and patient, servicer user preferences, values and needs. The process of achieving an agreement about what this looks and feels like is often referred to as shared decision making. The EBP process is often presented through five basic steps: (1) formulating the clinical question, (2) searching efficiently for the best available evidence, (3) critically analysing evidence for its validity and usefulness, (4) integrating the appraisal with personal clinical expertise and clients’ preferences, and (5) evaluating one’s performance or outcomes of actions [9].

### Evidence-based research (EBR)—justifying the research

Health research should build systematically on previous research [3, 4]. Despite this intentional basic principle, numerous studies indicate that researchers fail to use a systematic methodology to refer to earlier research when justifying and planning new research [10–13]. The term “evidence-based research” (EBR) was introduced to indicate the approach that is needed to reduce this inadvisable practice, which is an important source of research waste, and worse, it may lead to unnecessary harm for patients and study participants [14]. A process of EBR has been proposed [14] emphasising that researchers have a responsibility to plan and conduct new research informed by all former and ongoing research relevant to the proposed new research. Lund et al. highlight two responsibilities for research stakeholders, in order to meet the aims of EBR. Firstly, no new studies should be planned without adequate systematic review of existing evidence, showing the new research to be justified. Secondly, systematic reviews should be efficiently produced, updated and published. In a paper addressing “research waste”, Chalmers and Glasziou argued that new research should not be done unless the research questions addressed cannot be answered satisfactorily with existing evidence at the time [15], which aligns with the EBR idea. Identifying needs and establishing uncertainty as well as relevance of research minimises wasting research funds, and combines and supports the concepts of EBP and EBR.

### Public involvement in health research

Setting research agendas has, for a long time, been in the health researchers’ and funders domain. It may be criticized that researchers are influenced by research institutions and funders necessity to produce research that is highly cited and of high impact in academic journals, and that this may lead to research that may not necessarily reflect the research needs of patients or health professionals and other stakeholders that may benefit from research. There is increasing evidence of a mismatch between users of the research preferences and priorities (patients/clinicians) and those of researchers [5] and the UK James Lind Alliance has pioneered models of public involvement in research priority setting to address this mismatch [16].

Partnering with or involving a wide range of stakeholders in health and social care research (from developing research questions to input to design and delivery) is an accepted approach in many countries, with more emphasis on how to do this well [17]. For some, this is not just a utilitarian argument about improving research but also a way to improve research governance, (especially with publicly funded research), relevance and impact. The moral argument advocates that involvement is a right, because citizens should have a voice in publicly funded research, simply based on the saying ‘nothing about me without me’ [18].

In 1991, a strategy for UK National Health Service research and development acknowledged the role of different stakeholders in research. There followed enterprises, guidance and opportunities for increased patient and public involvement in research, for example the UK Clinical Research Network, which led on a programme of public involvement in research. The charitable sector was also developing approaches and initiatives to public involvement in their research, for example the Quality Research in Dementia (QRD) Programme at the Alzheimer’s Society [19]. In Norway, initiatives have been taken to promote user involvement in research, both through strategic processes and decisions and through user involvement in research projects. In 2011, the Government's National plan for health and care [20] emphasized that "Increased user involvement in research and innovation must be facilitated”, and that “User involvement ensures that new research is relevant for users and that new innovative solutions are in line with users' needs”. In the following, commitment to user involvement has been included in the HelseOmsorg 21-strategy [21], National guidelines [22] on user involvement have been published, and there has been an increasing advocacy for user involvement in state-funded research, initiated by the Research Council of Norway.

Fast forward to 2020 and there is a rich landscape of networks, co-ordinating and supportive organisations across the world dedicated to high quality public involvement in research (e.g., in the US; PCORI, and in the UK; National Institute for Health Research Centre for Engagement and Dissemination). There are numerous guides and toolkits, for example the GRIPP checklist [23], superseded by GRIPP2 [24] for reporting involvement in research and more recently the UK Standards for Public Involvement [25]. What tends to occupy people now is methods that involve people in authentic and meaningful ways, achieving diversity and inclusion in involvement, and assessing the impact and effect of public involvement in research.

Outputs from public involvement in research can be broad from improving research processes (e.g., study recruitment, data collection and dissemination) to better research information (e.g., consent forms) to ensuring that research is relevant for patients and the public.

### Needs led research (NLR)—relevant and useful

To increase its relevance and usefulness, new research should be aimed at answering evidence gaps based on a systematic review of existing evidence *and* the needs and priorities from users and society—we have called this “Needs Led Research” (NLR). To achieve NLR requires a thorough stepwise process to identify and prioritize evidence gaps that involves researchers, user groups and stakeholders. The process is typically undertaken in several steps and will provide guidance on which research to prioritize.

NLR should be of interest to many stakeholders, including researchers, institutions, non-governmental organizations (NGOs), funders, and health and social care policy makers. Dependant on the role of the initiator, different methods and measures might be used for a NLR process. However, there are some key steps, including (a) defining a scope, (b) gather evidence gaps, (c) prioritize evidence gaps, and (d) choose which evidence gap(s) to answer. The use of available public data, existing evidence and different kinds of user involvement can be relevant in all these steps. The methods and measures will depend on the available resources, contractual funding, and time, experience and knowledge of the team. Pitfalls threatening the validity of the final result of each step in the process should be carefully considered and described. The Dementia priority setting partnership with the James Lind Alliance is an excellent example of needs led research in the UK [26].

In 2017, the Kavli Trust, a Norwegian funder of health research, launched a new funding program aiming to fund relevant and needed research in an effort to reduce the disturbing amount of research waste. The principles of needs-led research were used. The Kavli Trust decided, based on an evaluation of research grants and the burden of disease in Norway, to fund research targeting selected evidence gaps within children and adolescent mental health. The evidence gaps were selected in a two-step process. First, experts identified actual evidence gaps through comprehensive literature searches, and second, users (patients, carers/parents and health professionals) prioritized the identified evidence gaps. The call for research included the top ten evidence gaps, and potential applicants had to address one or more of the selected evidence gaps to be eligible for funding from the Kavli Trust Programme on Health Research. Currently, ten on-going research projects have received grants through the program and conduct research to close some of the evidence gaps within this field.

The Faculty of Health Sciences at the Oslo Metropolitan University has also committed to NLR. Through the Bridgebuilder Initiative, they aim to link research priorities to health education and clinical practice to ensure that the new studies are relevant and useful for patients and society. Training in the principles of needs-led research and a profound involvement of users through a Ph.D. course provide guidance on the choice of topic and selection of research question(s). The initiative was influenced in part by the UK James Lind Alliance [16], which has pioneered models of public involvement in research priority setting. Fleurence and Torgerson state that setting priorities for research is necessary to secure efficient use of limited resources, but that the usage of such methods by researchers and commissioners of research have been limited [27]. Hence, the methodology should, according to Fleurence and Torgerson meet the objectives of the health system; “*to provide the most health benefits to the population that it serves within the budget constraint and while respecting equity considerations”.* According to Kok and co-workers it is important to support research that meets locally expressed needs involving people embedded in the contexts in which the results can be used. They emphasize that *“involvement of health sector professionals in the design, conduct and interpretation of research appears to be an especially worthwhile investment*” [28].

In the Bridgebuilder Initiative [29] Ph.D.-students from various health professions have applied different methods and approaches to involve users and stakeholders to decide what their research should be focusing on and which research questions that could and should be answered based on NLR principles. This represents a considerable change in how Ph.D. studies in health are conducted and organised at Oslo Metropolitan University in Norway. Training and support for user involvement in NLR processes was provided and taken forward by students and supervisors. Currently, eight Ph.D.-students are well underway in conducting research that will provide new knowledge addressing evidence gaps considered particularly important and needed by the users. After finalising the NLR-process, users have also been included as user representatives in the research projects, to follow-up on the Bridgebuilder Projects’ commitment to user involvement. The Ph.D. projects are based in hospital settings, such as intensive care, stroke management and maternity care, as well as community-based settings including elderly care and services for children and youth.

### Systematic reviews of existing evidence—the foundation for EBP, EBR and NLR

As described above, clinical decisions should be based on a synthesis of the excising relevant evidence. For evidence-based decisions—EBP—clinicians often search for information and sources from higher levels of an evidence pyramid [30], such as clinical guidelines and synthesised summaries and systems.

A literature search for systematic reviews constitutes a starting point for EBR enabling students to “get to know” the available research within the field of interest—the scope. Furthermore, we argue that the *sequence* of searches for evidence gaps (from systematic reviews) or unknowns from stakeholders is of minor importance. However, both aspects are needed and covered in the process. This is described in more detail below.

A systematic review is a review of a clearly formulated question that uses systematic and reproducible methods to identify, select and critically appraise all relevant research, and to collect and analyze data from the studies that are included in the review. Through the identification of systematic reviews, researchers will get an overview of all existing evidence on a given topic and thereby also information about evidence gaps and the need for new studies. Systematic reviews of the effect of interventions are most common, but reviews concerning other types of research questions also exist. A systematic review of effect of interventions might include a meta-analysis, which is a statistical summary of results from many primary studies providing a common estimate of effect. More often, effect estimates for specific comparisons and outcomes from systematic reviews are presented by its quality of evidence using the GRADE approach [8]. Furthermore, reviews summarizing results from qualitative studies is a growing field. The latter may originate from different methods and have different names; however, *meta-synthesis* is often used as an umbrella term.

## The Bridge Building Model

**Fig. 1:**
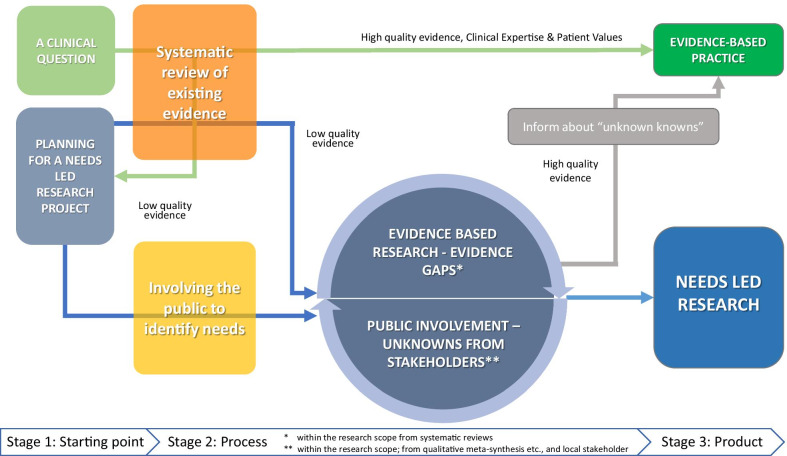
The starting point for the process may be from different origins, anchoring (the primary organization driving it) and aims. We describe two points of entrance in the model

We have combined the approaches and principles behind EBP, EBR and NLR and public involvement into a model to visualise their inherent links. The model presented in Fig. 1, outlines a process from a more or less well-defined scope within health, via different pathways and actions to result in Needs Led Research that may influence Evidence Based Practice.

1. ***A clinical question—the starting point for EBP***
  The starting point is a clinical setting aiming to identify updated evidence for a clinical question. The literature search should be based on a *clearly focused question* and concentrate on “the best available evidence”, preferably from systematic reviews or clinical guidelines. If there is lack of systematic reviews to answer the clinical question, it is recommended that the clinician/researcher may consider conducting their own review of the evidence (Lund et al. 2016). Further, it might be that there is even lack of primary and good quality studies on the topic in question. If so, a knowledge gap has probably been identified, and there is a need for new studies. This might thus be a starting point for a new research project, as described below.
2. ***An intention to involve stakeholders in research priorities—the starting point for NLR***
  The starting point is an aim to carry out a research project within a particular scope, in which user involvement will be embraced. The first and most important decision in preparing any research is to determine its focus by framing the question(s) the research seeks to answer. In the case of NLR however, we start with a broad enough scope to ensure that the users’ preferences will influence the outcome of the process, i.e., the final research questions to be explored. It is important that user involvement process is not tokenistic whereby the researchers have already decided what their research questions will be, and are not open to comment or critique of their ideas.

### Putting EBP into practice

EBP combines insights from *existing evidence* (e.g., systematic reviews) with *clinical reasoning* based on one’s *clinical expertise and experience* and patient preference, values and beliefs in order to adapt care and improve outcomes for *individual patients*. This requires patients to be part of conversations about alternatives and clinical options.

Although patient input into decisions about their care is respected and desired, there might be some barriers, such as the clinician’s lack of time and knowledge, and sociocultural features such as gender, race, and age [7]. The most important thing is to listen to the patient, this requires interpersonal skills, and whilst such skills can be learned, tools can also be helpful. The Ask Share Know (ASK) Model [31] clarifies three questions that patients and families should ask their healthcare providers in order to obtain the information needed for shared decision-making. (1) What are my options? (2) What are the possible benefits and harms of those options? (3) How likely are each of those benefits and harms to happen to me, and what will happen if I do nothing?

More details about how to incorporate clinical judgment and patient preferences into evidence-based decision-making is outside the scope of this paper. This subject area has, however, been written well about in much more details elsewhere [32–35].

### Putting NLR into practice

A NLR process can be initiated from researchers, clinicians, research organisations, user organisations or stakeholders or others, with different groups involved providing input and playing key roles in the decision-making steps of the process.

First, a scope for the NLR must be defined, and dependant on the field of interest this scope may be wide or narrow. Typically, the more research available the narrower the scope must be to make the following steps of the process feasible. The scope can be derived from the researcher or research groups interest and experience or from the user organisation or stakeholders stated purpose and actions.

When the scope is defined, the search for evidence gaps begins. The steps include user involvement and systematic literature searches, but their order and extensiveness may vary. Researchers and librarians conduct a systematic search for systematic reviews within the scope of interest. These reviews may identify further research questions, where evidence is lacking, or is out of date. The identified evidence gaps should be evaluated by users and stakeholders, and assessed for their relevance to needs and importance. Furthermore, users and stakeholders may suggest additional topics or questions for the researchers to consider.

User groups and stakeholders are invited to suggest and discuss their needs within the scope of interest. This process results in research topics that are of particular interest to users and stakeholders. Researchers search the existing literature to evaluate whether the suggested evidence gaps are true evidence gaps or if they represent “*unknown knowns*”—i.e., questions for which research evidence exist, but these evidences are unknown to the users. Evidence about “*unknown knowns*” should be communicated to the clinical society and users, and should not be the topic of future research.

Gathering questions about needs from a variety of stakeholders may require different approaches, but the most important aspect is that a wide range of relevant contributors take part in this. It is also important that they feel confident and empowered to communicate their needs. Common methods include online and paper surveys, discussion (focus) groups and 1:1 conversation. Working with stakeholder groups such as patient charities can short cut the process, as they will already have established communities that can be approached. Choosing anonymous routes for feedback about clinical or patient needs helps contributors know that their questions will not be attributed and may encourage more candour. Barriers to people taking part need to be considered, such as language, cultural issues, limitations in literacy or access to taking part. The most accessible approach is to encourage people to write or say what they want to, rather than asking them to fit their contribution in to a fixed model such as the PICO (Population, Intervention, Comparison and Outcome) model for clinical questions [36]. However, this requires teams to consider carefully the resource they have to support the interpretation of ‘free text’. Using a framework can also be helpful, such as asking people to think about their needs in terms of cause, care and cure or diagnosis, treatment and prognosis for health research. Stakeholders should always be thanked for their contribution and kept up to date with progress in the NLR process.

To sum up on NLR, to conduct *thorough and systematic searches for systematic reviews*, and to use *verified methods of user involvement*, will help to ensure that our research questions are *previously unanswered* and *relevant* to patients, relatives and healthcare professionals. That is, the research is needs identified, or needs led, in a double sense.

## Conclusions

Evidence-based practice, evidence-based research, public involvement and needs led research have all been highlighted as important, but there has been a lack of integration. Based on an aim to capitalise on the joining of the rationale and methods for these approaches, we have hereby presented a model for incorporation—the Bridge Building Model. We think the model may help health professionals, researchers, and funders to work in a more effectively way by bridging health and social care needs identification, evidence-based practice and research. This model is currently being used in the Bridgebuilder Initiative, learning and experiences thereof will be published in the future.

We recommend both clinicians and researchers to carefully think through the justification and procedures incorporated in the Bridge Building Model and to try out this model as a successful way of working.

## Data Availability

Not applicable.

## Abbreviations

EBP: Evidence Based Practice
EBM: Evidence Based Medicine
NLR: Needs Led Research

## References

[1] Guyatt GH (1991). Evidence-based medicine. ACP J Club.

[2] Dawes M, Summerskill W, Glasziou P, Cartabellotta A, Martin J, Hopayian K (2005). Sicily statement on evidence-based practice. BMC Med Educ.

[3] Young C, Horton R (2005). Putting clinical trials into context. Lancet.

[4] Chalmers I (2005). Academia's failure to support systematic reviews. Lancet.

[5] Crowe S, Fenton M, Hall M, Cowan K, Chalmers I (2015). Patients', clinicians' and the research communities' priorities for treatment research: there is an important mismatch. Res Involv Engagem.

[6] Sackett DL (1997). Evidence-based medicine. Semin Perinatol.

[7] Lin SH, Murphy SL, Robinson JC (2010). Facilitating evidence-based practice: process, strategies, and resources. Am J Occup Ther.

[8] Neumann I, Santesso N, Akl EA, Rind DM, Vandvik PO, Alonso-Coello P (2016). A guide for health professionals to interpret and use recommendations in guidelines developed with the GRADE approach. J Clin Epidemiol.

[9] Straus SE, Richardson WS, Glasziou P, Haynes RB (2005). Evidence-based medicine.

[10] Clarke M, Hopewell S, Chalmers I (2010). Clinical trials should begin and end with systematic reviews of relevant evidence: 12 years and waiting. Lancet.

[11] Sheth U, Simunovic N, Tornetta P, Einhorn TA, Bhandari M (2011). Poor citation of prior evidence in hip fracture trials. J Bone Jt Surg Am.

[12] Habre C, Tramèr MR, Pöpping DM, Elia N (2014). Ability of a meta-analysis to prevent redundant research: systematic review of studies on pain from propofol injection. BMJ.

[13] Sawin VI, Robinson KA (2016). Biased and inadequate citation of prior research in reports of cardiovascular trials is a continuing source of waste in research. J Clin Epidemiol.

[14] Lund H, Brunnhuber K, Juhl C, Robinson K, Leenaars M, Dorch BF (2016). Towards evidence based research. BMJ.

[15] Chalmers I, Glasziou P (2009). Avoidable waste in the production and reporting of research evidence. Lancet.

[16] Partridge N, Scadding J (2004). The James Lind Alliance: patients and clinicians should jointly identify their priorities for clinical trials. Lancet.

[17] Concannon TW, Grant S, Welch V, Petkovic J, Selby J, Crowe S (2019). Practical guidance for involving stakeholders in health research. J Gen Intern Med.

[18] Delbanco T, Berwick DM, Boufford JI, Edgman-Levitan S, Ollenschläger G, Plamping D (2001). Healthcare in a land called PeoplePower: nothing about me without me. Health Expect.

[19] S W-CBaN. Alzheimers society. A history of the research network. 2015.

[20] omsorgsdepartementet. H-o. Nasjonal helse- og omsorgsplan: 2011–2015. Oslo2011. https://www.regjeringen.no/no/dokumenter/meld-st-16-20102011/id639794/.

[21] Services MoHaC. HelseOmsorg 21. 2014.

[22] service Npfctritsh. National Guidelines for user involvement in health research in hospital care. 2020.

[23] Staniszewska S, Brett J, Mockford C, Barber R (2011). The GRIPP checklist: strengthening the quality of patient and public involvement reporting in research. Int J Technol Assess Health Care.

[24] Staniszewska S, Brett J, Simera I, Seers K, Mockford C, Goodlad S (2017). GRIPP2 reporting checklists: tools to improve reporting of patient and public involvement in research. BMJ.

[25] Crowe S, Adebajo A, Esmael H, Denegri S, Martin A, McAlister B (2020). 'All hands-on deck', working together to develop UK standards for public involvement in research. Res Involv Engagem.

[26] Kelly S, Lafortune L, Hart N, Cowan K, Fenton M, Brayne C (2015). Dementia priority setting partnership with the James Lind Alliance: using patient and public involvement and the evidence base to inform the research agenda. Age Ageing.

[27] Fleurence RL, Torgerson DJ (2004). Setting priorities for research. Health Policy.

[28] Kok MO, Gyapong JO, Wolffers I, Ofori-Adjei D, Ruitenberg J (2016). Which health research gets used and why? An empirical analysis of 30 cases. Health Res Policy Syst.

[29] University OM. The Bridgebulider initiative 2021. https://www.oslomet.no/om/hv/fou/brobyggersatsingen.

[30] Haynes RB (2006). Of studies, syntheses, synopses, summaries, and systems: the "5S" evolution of information services for evidence-based health care decisions. ACP J Club.

[31] Excellence NfCfR. Ask Share Know. 2021.

[32] Mühlbacher AC, Juhnke C (2013). Patient preferences versus physicians' judgement: does it make a difference in healthcare decision making?. Appl Health Econ Health Policy.

[33] Kaplan RM, Frosch DL (2005). Decision making in medicine and health care. Annu Rev Clin Psychol.

[34] Salmi LR, Côté P, Cedraschi C (2020). Covering patient's perspective in case-based critical review articles to improve shared decision making in complex cases. Health Expect.

[35] Djulbegovic B, Guyatt GH (2017). Progress in evidence-based medicine: a quarter century on. Lancet.

[36] Tovey, D. How to clarify a clinical question. BMJ Best Pract. 2021.

